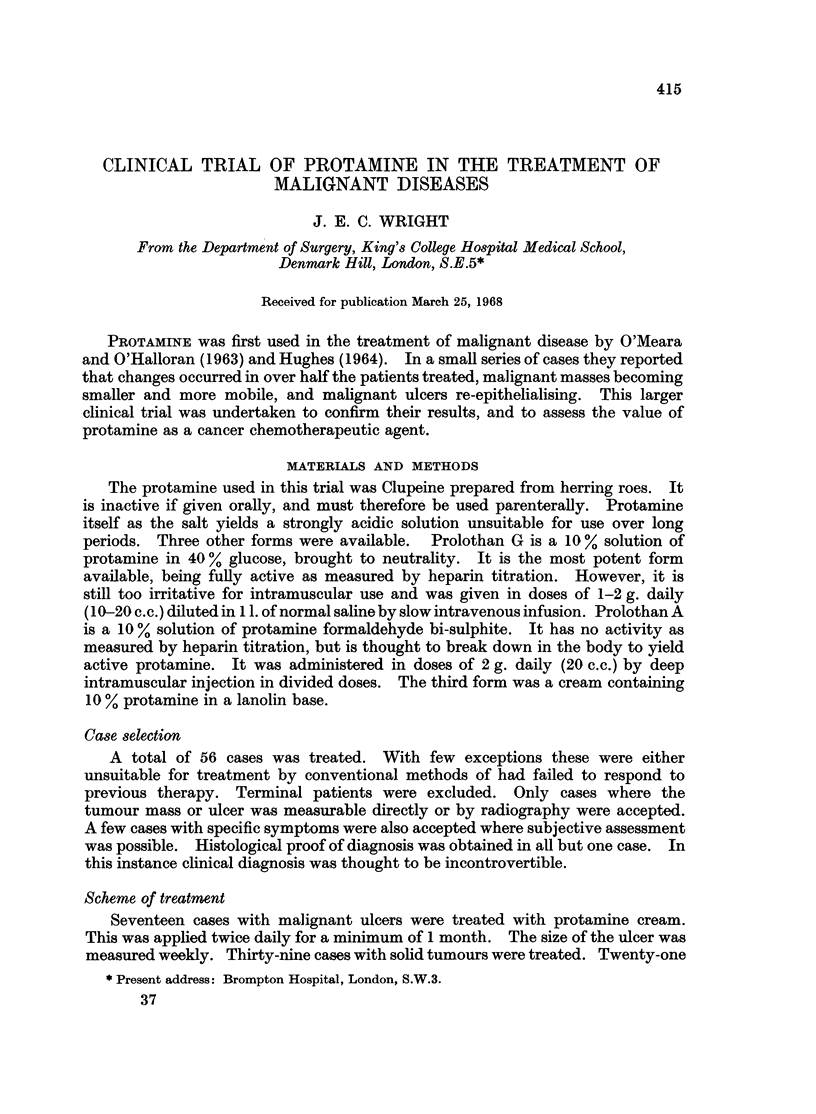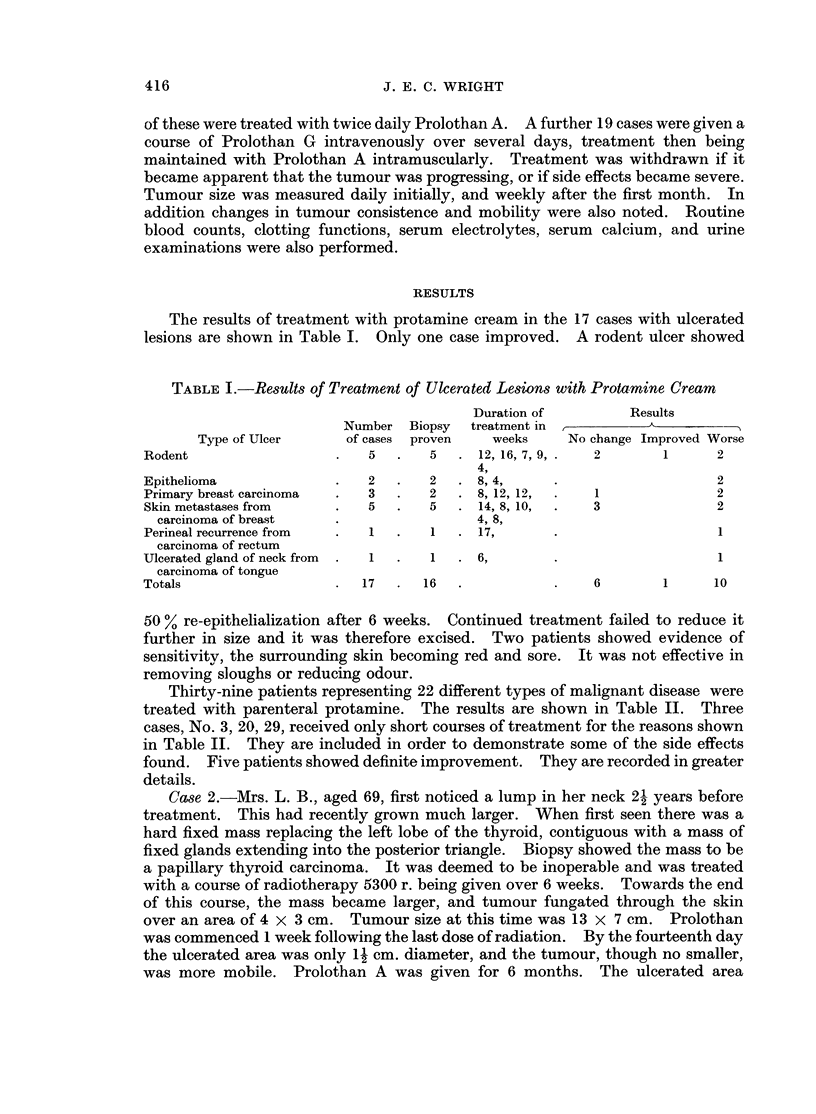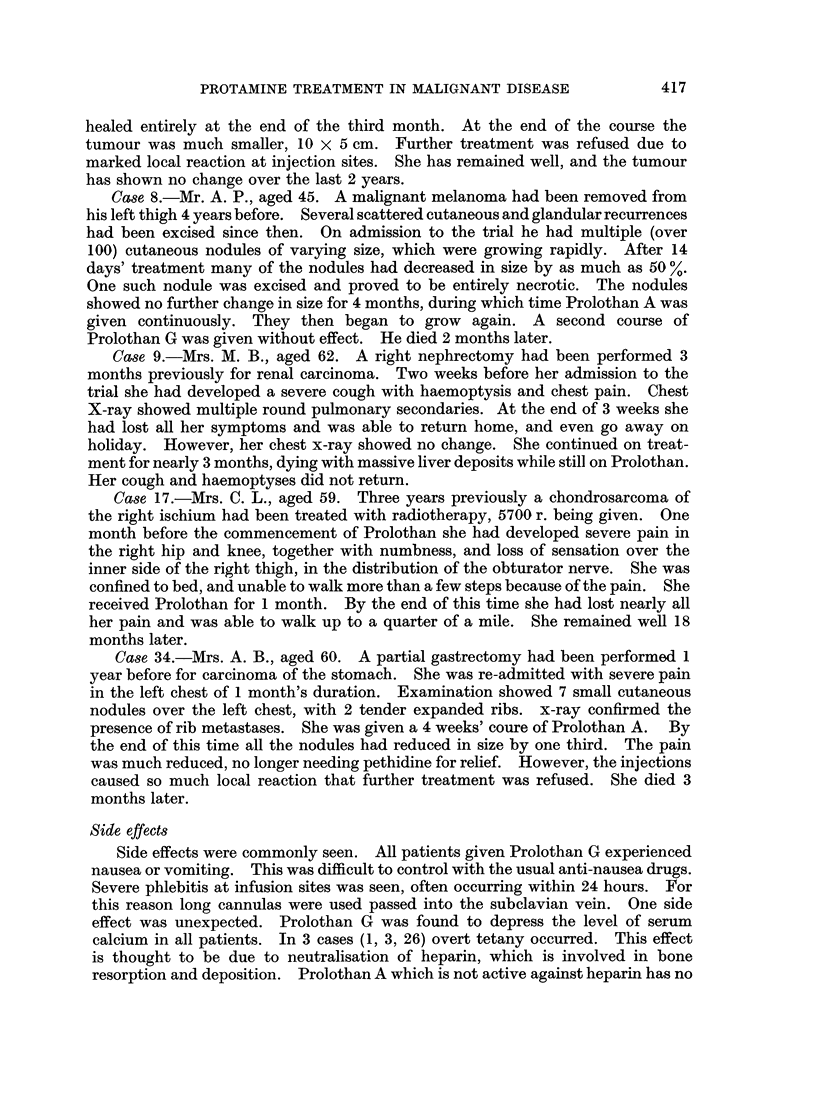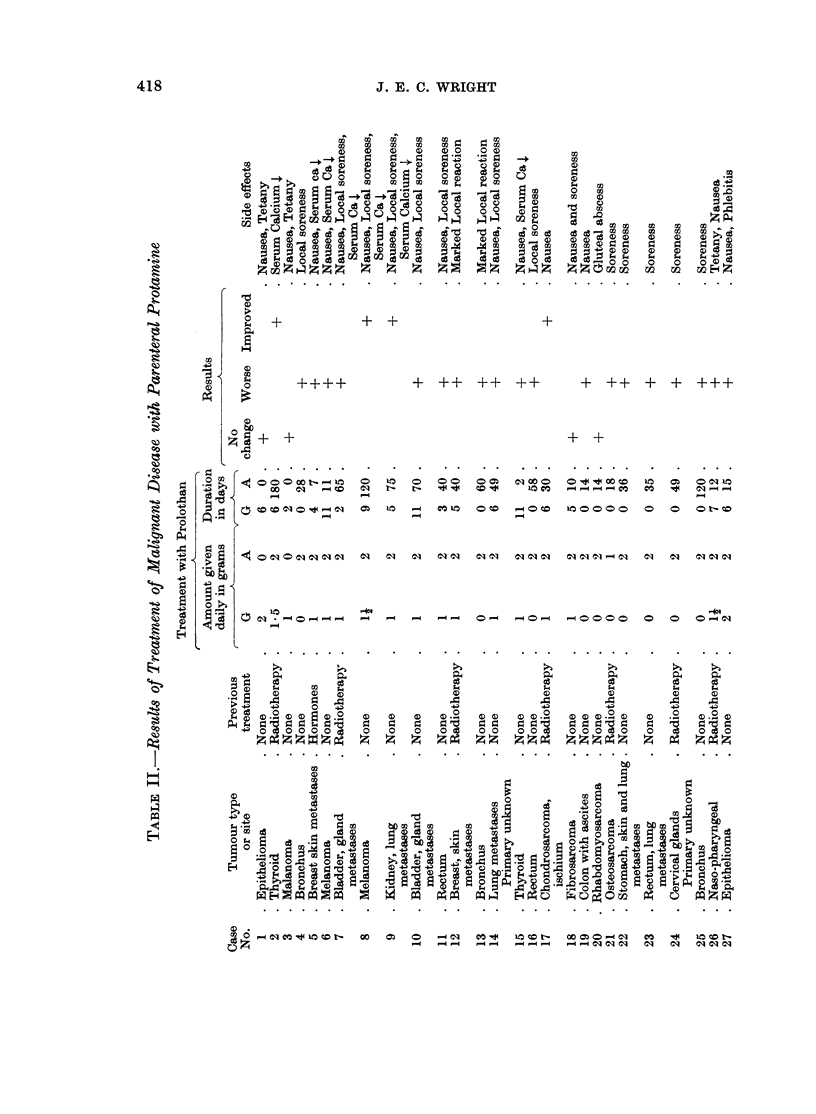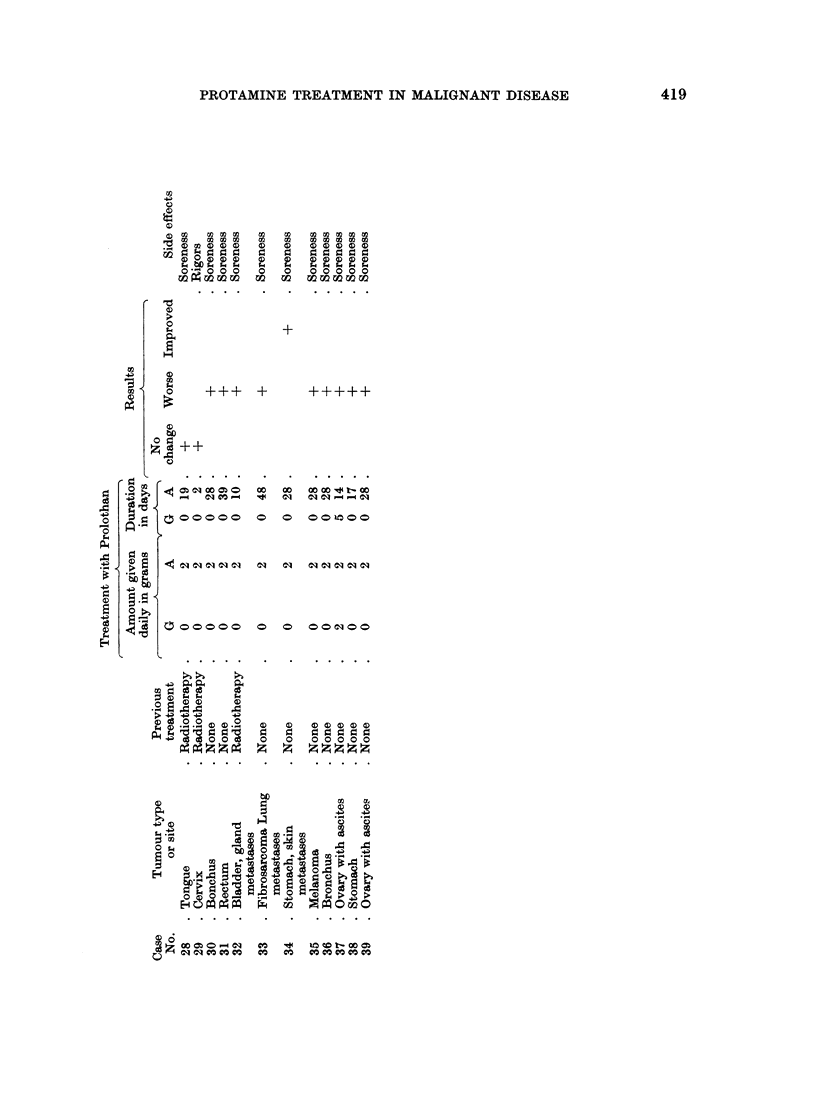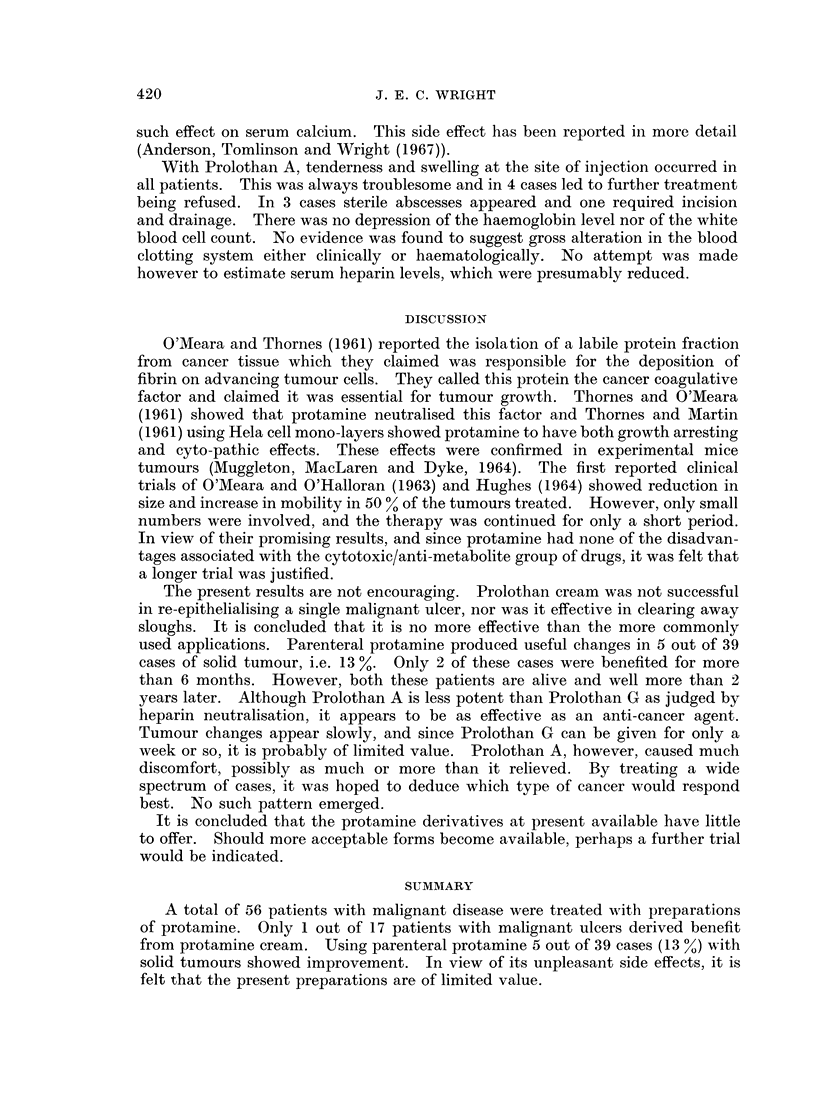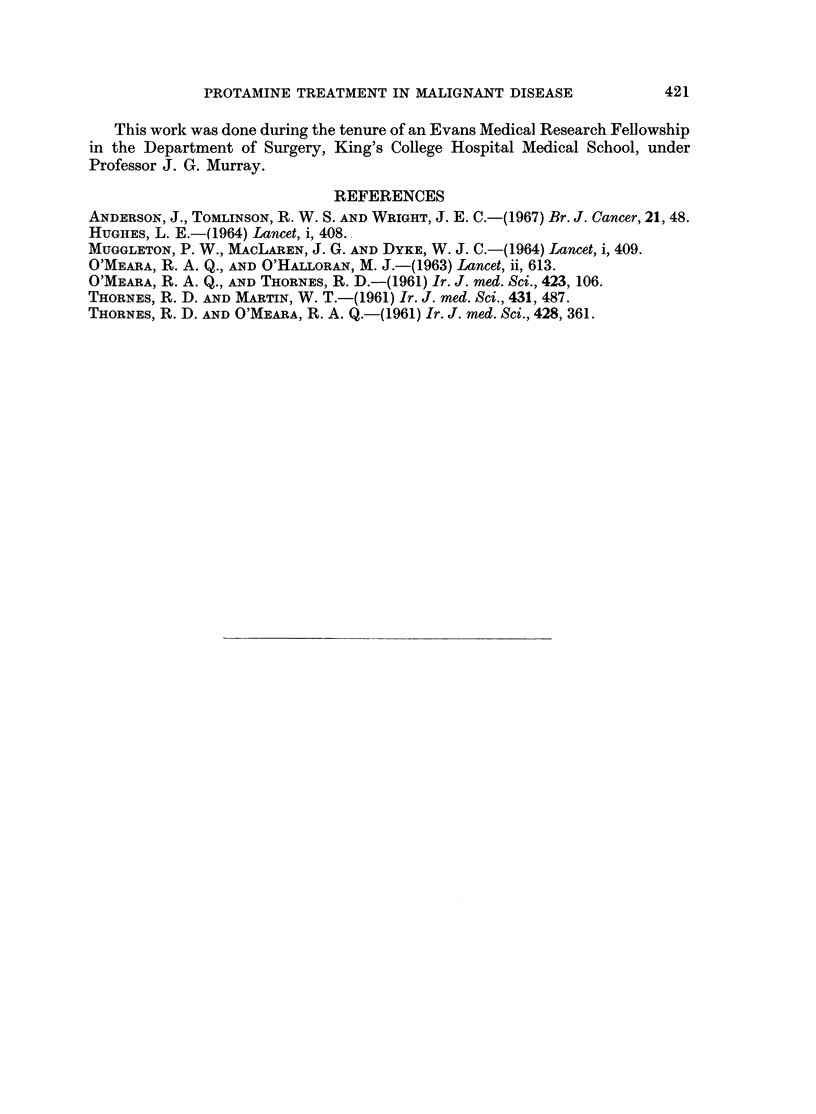# Clinical trial of protamine in the treatment of malignant diseases.

**DOI:** 10.1038/bjc.1968.49

**Published:** 1968-09

**Authors:** J. E. Wright


### CLINICAL TRIAL OF PROTAMINE IN THE TREATMENT OF MALIGNANT DISEASES

J. E. C. WRIGHT

*From the Department of Surgery, King's College Hospital Medical School, Denmark Hill, London, S.E.5**

Received for publication March 25, 1968

PROTAMINE was first used in the treatment of malignant disease by O'Meara and O'Halloran (1963) and Hughes (1964). In a small series of cases they reported that changes occurred in over half the patients treated, malignant masses becoming smaller and more mobile, and malignant ulcers re-epithelialising. This larger clinical trial was undertaken to confirm their results, and to assess the value of protamine as a cancer chemotherapeutic agent.

### MATERIALS AND METHODS

The protamine used in this trial was Clupeine prepared from herring roes. It is inactive if given orally, and must therefore be used parenterally. Protamine itself as the salt yields a strongly acidic solution unsuitable for use over long periods. Three other forms were available. Prolothan G is a 10% solution of protamine in 40% glucose, brought to neutrality. It is the most potent form available, being fully active as measured by heparin titration. However, it is still too irritative for intramuscular use and was given in doses of 1–2 g. daily (10–20 c.c.) diluted in 1 l. of normal saline by slow intravenous infusion. Prolothan A is a 10% solution of protamine formaldehyde bi-sulphite. It has no activity as measured by heparin titration, but is thought to break down in the body to yield active protamine. It was administered in doses of 2 g. daily (20 c.c.) by deep intramuscular injection in divided doses. The third form was a cream containing 10% protamine in a lanolin base.

### *Case selection*

A total of 56 cases was treated. With few exceptions these were either unsuitable for treatment by conventional methods of had failed to respond to previous therapy. Terminal patients were excluded. Only cases where the tumour mass or ulcer was measurable directly or by radiography were accepted. A few cases with specific symptoms were also accepted where subjective assessment was possible. Histological proof of diagnosis was obtained in all but one case. In this instance clinical diagnosis was thought to be incontrovertible.

### *Scheme of treatment*

Seventeen cases with malignant ulcers were treated with protamine cream. This was applied twice daily for a minimum of 1 month. The size of the ulcer was measured weekly. Thirty-nine cases with solid tumours were treated. Twenty-one

* Present address: Brompton Hospital, London, S.W.3.

of these were treated with twice daily Prolothan A. A further 19 cases were given a course of Prolothan G intravenously over several days, treatment then being maintained with Prolothan A intramuscularly. Treatment was withdrawn if it became apparent that the tumour was progressing, or if side effects became severe. Tumour size was measured daily initially, and weekly after the first month. In addition changes in tumour consistence and mobility were also noted. Routine blood counts, clotting functions, serum electrolytes, serum calcium, and urine examinations were also performed.

### RESULTS

The results of treatment with protamine cream in the 17 cases with ulcerated lesions are shown in Table I. Only one case improved. A rodent ulcer showed

TABLE I.—*Results of Treatment of Ulcerated Lesions with Protamine Cream*

| Type of Ulcer | Number of cases | Biopsy proven | Duration of treatment in weeks | Results: No change | Improved | Worse |
|---|---|---|---|---|---|---|
| Rodent | 5 | 5 | 12, 16, 7, 9, 4, | 2 | 1 | 2 |
| Epithelioma | 2 | 2 | 8, 4, | | | 2 |
| Primary breast carcinoma | 3 | 2 | 8, 12, 12, | 1 | | 2 |
| Skin metastases from carcinoma of breast | 5 | 5 | 14, 8, 10, 4, 8, | 3 | | 2 |
| Perineal recurrence from carcinoma of rectum | 1 | 1 | 17, | | | 1 |
| Ulcerated gland of neck from carcinoma of tongue | 1 | 1 | 6, | | | 1 |
| Totals | 17 | 16 | | 6 | 1 | 10 |

50 % re-epithelialization after 6 weeks. Continued treatment failed to reduce it further in size and it was therefore excised. Two patients showed evidence of sensitivity, the surrounding skin becoming red and sore. It was not effective in removing sloughs or reducing odour.

Thirty-nine patients representing 22 different types of malignant disease were treated with parenteral protamine. The results are shown in Table II. Three cases, No. 3, 20, 29, received only short courses of treatment for the reasons shown in Table II. They are included in order to demonstrate some of the side effects found. Five patients showed definite improvement. They are recorded in greater details.

*Case* 2.—Mrs. L. B., aged 69, first noticed a lump in her neck $2\frac{1}{2}$ years before treatment. This had recently grown much larger. When first seen there was a hard fixed mass replacing the left lobe of the thyroid, contiguous with a mass of fixed glands extending into the posterior triangle. Biopsy showed the mass to be a papillary thyroid carcinoma. It was deemed to be inoperable and was treated with a course of radiotherapy 5300 r. being given over 6 weeks. Towards the end of this course, the mass became larger, and tumour fungated through the skin over an area of $4 \times 3$ cm. Tumour size at this time was $13 \times 7$ cm. Prolothan was commenced 1 week following the last dose of radiation. By the fourteenth day the ulcerated area was only $1\frac{1}{2}$ cm. diameter, and the tumour, though no smaller, was more mobile. Prolothan A was given for 6 months. The ulcerated area healed entirely at the end of the third month. At the end of the course the tumour was much smaller, 10 × 5 cm. Further treatment was refused due to marked local reaction at injection sites. She has remained well, and the tumour has shown no change over the last 2 years.

*Case* 8.—Mr. A. P., aged 45. A malignant melanoma had been removed from his left thigh 4 years before. Several scattered cutaneous and glandular recurrences had been excised since then. On admission to the trial he had multiple (over 100) cutaneous nodules of varying size, which were growing rapidly. After 14 days' treatment many of the nodules had decreased in size by as much as 50%. One such nodule was excised and proved to be entirely necrotic. The nodules showed no further change in size for 4 months, during which time Prolothan A was given continuously. They then began to grow again. A second course of Prolothan G was given without effect. He died 2 months later.

*Case* 9.—Mrs. M. B., aged 62. A right nephrectomy had been performed 3 months previously for renal carcinoma. Two weeks before her admission to the trial she had developed a severe cough with haemoptysis and chest pain. Chest X-ray showed multiple round pulmonary secondaries. At the end of 3 weeks she had lost all her symptoms and was able to return home, and even go away on holiday. However, her chest x-ray showed no change. She continued on treatment for nearly 3 months, dying with massive liver deposits while still on Prolothan. Her cough and haemoptyses did not return.

*Case* 17.—Mrs. C. L., aged 59. Three years previously a chondrosarcoma of the right ischium had been treated with radiotherapy, 5700 r. being given. One month before the commencement of Prolothan she had developed severe pain in the right hip and knee, together with numbness, and loss of sensation over the inner side of the right thigh, in the distribution of the obturator nerve. She was confined to bed, and unable to walk more than a few steps because of the pain. She received Prolothan for 1 month. By the end of this time she had lost nearly all her pain and was able to walk up to a quarter of a mile. She remained well 18 months later.

*Case* 34.—Mrs. A. B., aged 60. A partial gastrectomy had been performed 1 year before for carcinoma of the stomach. She was re-admitted with severe pain in the left chest of 1 month's duration. Examination showed 7 small cutaneous nodules over the left chest, with 2 tender expanded ribs. x-ray confirmed the presence of rib metastases. She was given a 4 weeks' coure of Prolothan A. By the end of this time all the nodules had reduced in size by one third. The pain was much reduced, no longer needing pethidine for relief. However, the injections caused so much local reaction that further treatment was refused. She died 3 months later.

*Side effects*

Side effects were commonly seen. All patients given Prolothan G experienced nausea or vomiting. This was difficult to control with the usual anti-nausea drugs. Severe phlebitis at infusion sites was seen, often occurring within 24 hours. For this reason long cannulas were used passed into the subclavian vein. One side effect was unexpected. Prolothan G was found to depress the level of serum calcium in all patients. In 3 cases (1, 3, 26) overt tetany occurred. This effect is thought to be due to neutralisation of heparin, which is involved in bone resorption and deposition. Prolothan A which is not active against heparin has no

TABLE II.—*Results of Treatment of Malignant Disease with Parenteral Protamine*

| Case No. | Tumour type or site | Previous treatment | Treatment with Prolothan: Amount given daily in grams G | A | Duration in days G | A | Results: No change | Worse | Improved | Side effects |
|---|---|---|---|---|---|---|---|---|---|---|
| 1 | Epithelioma | None | 2 | 0 | 6 | 0 | + | | | Nausea, Tetany |
| 2 | Thyroid | Radiotherapy | 1·5 | 2 | 6 | 180 | | | + | Serum Calcium ↓ |
| 3 | Malanoma | None | 1 | 0 | 2 | 0 | + | | | Nausea, Tetany |
| 4 | Bronchus | None | 0 | 2 | 0 | 28 | | + | | Local soreness |
| 5 | Breast skin metastases | Hormones | 1 | 2 | 4 | 7 | | + | | Nausea, Serum ca ↓ |
| 6 | Melanoma | None | 1 | 2 | 11 | 11 | | + | | Nausea, Serum Ca ↓ |
| 7 | Bladder, gland metastases | Radiotherapy | 1 | 2 | 2 | 65 | | + | | Nausea, Local soreness, Serum Ca ↓ |
| 8 | Melanoma | None | 1½ | 2 | 9 | 120 | | | + | Nausea, Local soreness, Serum Ca ↓ |
| 9 | Kidney, lung metastases | None | 1 | 2 | 5 | 75 | | | + | Nausea, Local soreness, Serum Calcium ↓ |
| 10 | Bladder, gland metastases | None | 1 | 2 | 11 | 70 | | + | | Nausea, Local soreness |
| 11 | Rectum | None | 1 | 2 | 3 | 40 | | + | | Nausea, Local soreness |
| 12 | Breast, skin metastases | Radiotherapy | 1 | 2 | 5 | 40 | | + | | Marked Local reaction |
| 13 | Bronchus | None | 0 | 2 | 0 | 60 | | + | | Marked Local reaction |
| 14 | Lung metastases Primary unknown | None | 1 | 2 | 6 | 49 | | + | | Nausea, Local soreness |
| 15 | Thyroid | None | 1 | 2 | 11 | 2 | | + | | Nausea, Serum Ca ↓ |
| 16 | Rectum | None | 0 | 2 | 0 | 58 | | + | | Local soreness |
| 17 | Chondrosarcoma, ischium | Radiotherapy | 1 | 2 | 6 | 30 | | | + | Nausea |
| 18 | Fibrosarcoma | None | 1 | 2 | 5 | 10 | + | | | Nausea and soreness |
| 19 | Colon with ascites | None | 0 | 2 | 0 | 14 | | + | | Nausea |
| 20 | Rhabdomyosarcoma | None | 0 | 2 | 0 | 14 | + | | | Gluteal abscess |
| 21 | Osteosarcoma | Radiotherapy | 0 | 1 | 0 | 18 | | + | | Soreness |
| 22 | Stomach, skin and lung metastases | None | 0 | 2 | 0 | 36 | | + | | Soreness |
| 23 | Rectum, lung metastases | None | 0 | 2 | 0 | 35 | | + | | Soreness |
| 24 | Cervical glands Primary unknown | Radiotherapy | 0 | 2 | 0 | 49 | | + | | Soreness |
| 25 | Bronchus | None | 0 | 2 | 0 | 120 | | + | | Soreness |
| 26 | Naso-pharyngeal | Radiotherapy | 1½ | 2 | 7 | 12 | | + | | Tetany, Nausea |
| 27 | Epithelioma | None | 2 | 2 | 6 | 15 | | + | | Nausea, Phlebitis |

| Case No. | Tumour type or site | Previous treatment | Treatment with Prolothan: Amount given daily in grams G | A | Duration in days G | A | Results: No change | Worse | Improved | Side effects |
|---|---|---|---|---|---|---|---|---|---|---|
| 28 | Tongue | Radiotherapy | 0 | 2 | 0 | 19 | + | | | Soreness |
| 29 | Cervix | Radiotherapy | 0 | 2 | 0 | 2 | + | | | Rigors |
| 30 | Bonchus | None | 0 | 2 | 0 | 28 | | + | | Soreness |
| 31 | Rectum | None | 0 | 2 | 0 | 39 | | + | | Soreness |
| 32 | Bladder, gland metastases | Radiotherapy | 0 | 2 | 0 | 10 | | + | | Soreness |
| 33 | Fibrosarcoma Lung metastases | None | 0 | 2 | 0 | 48 | | + | | Soreness |
| 34 | Stomach, skin metastases | None | 0 | 2 | 0 | 28 | | | + | Soreness |
| 35 | Melanoma | None | 0 | 2 | 0 | 28 | | + | | Soreness |
| 36 | Bronchus | None | 0 | 2 | 0 | 28 | | + | | Soreness |
| 37 | Ovary with ascites | None | 2 | 2 | 5 | 14 | | + | | Soreness |
| 38 | Stomach | None | 0 | 2 | 0 | 17 | | + | | Soreness |
| 39 | Ovary with ascites | None | 0 | 2 | 0 | 28 | | + | | Soreness |

such effect on serum calcium. This side effect has been reported in more detail (Anderson, Tomlinson and Wright (1967)).

With Prolothan A, tenderness and swelling at the site of injection occurred in all patients. This was always troublesome and in 4 cases led to further treatment being refused. In 3 cases sterile abscesses appeared and one required incision and drainage. There was no depression of the haemoglobin level nor of the white blood cell count. No evidence was found to suggest gross alteration in the blood clotting system either clinically or haematologically. No attempt was made however to estimate serum heparin levels, which were presumably reduced.

### DISCUSSION

O'Meara and Thornes (1961) reported the isolation of a labile protein fraction from cancer tissue which they claimed was responsible for the deposition of fibrin on advancing tumour cells. They called this protein the cancer coagulative factor and claimed it was essential for tumour growth. Thornes and O'Meara (1961) showed that protamine neutralised this factor and Thornes and Martin (1961) using Hela cell mono-layers showed protamine to have both growth arresting and cyto-pathic effects. These effects were confirmed in experimental mice tumours (Muggleton, MacLaren and Dyke, 1964). The first reported clinical trials of O'Meara and O'Halloran (1963) and Hughes (1964) showed reduction in size and increase in mobility in 50 % of the tumours treated. However, only small numbers were involved, and the therapy was continued for only a short period. In view of their promising results, and since protamine had none of the disadvantages associated with the cytotoxic/anti-metabolite group of drugs, it was felt that a longer trial was justified.

The present results are not encouraging. Prolothan cream was not successful in re-epithelialising a single malignant ulcer, nor was it effective in clearing away sloughs. It is concluded that it is no more effective than the more commonly used applications. Parenteral protamine produced useful changes in 5 out of 39 cases of solid tumour, i.e. 13 %. Only 2 of these cases were benefited for more than 6 months. However, both these patients are alive and well more than 2 years later. Although Prolothan A is less potent than Prolothan G as judged by heparin neutralisation, it appears to be as effective as an anti-cancer agent. Tumour changes appear slowly, and since Prolothan G can be given for only a week or so, it is probably of limited value. Prolothan A, however, caused much discomfort, possibly as much or more than it relieved. By treating a wide spectrum of cases, it was hoped to deduce which type of cancer would respond best. No such pattern emerged.

It is concluded that the protamine derivatives at present available have little to offer. Should more acceptable forms become available, perhaps a further trial would be indicated.

### SUMMARY

A total of 56 patients with malignant disease were treated with preparations of protamine. Only 1 out of 17 patients with malignant ulcers derived benefit from protamine cream. Using parenteral protamine 5 out of 39 cases (13 %) with solid tumours showed improvement. In view of its unpleasant side effects, it is felt that the present preparations are of limited value.

This work was done during the tenure of an Evans Medical Research Fellowship in the Department of Surgery, King's College Hospital Medical School, under Professor J. G. Murray.

### REFERENCES

ANDERSON, J., TOMLINSON, R. W. S. AND WRIGHT, J. E. C.—(1967) *Br. J. Cancer*, **21**, 48.
HUGHES, L. E.—(1964) *Lancet*, i, 408.
MUGGLETON, P. W., MACLAREN, J. G. AND DYKE, W. J. C.—(1964) *Lancet*, i, 409.
O'MEARA, R. A. Q., AND O'HALLORAN, M. J.—(1963) *Lancet*, ii, 613.
O'MEARA, R. A. Q., AND THORNES, R. D.—(1961) *Ir. J. med. Sci.*, **423**, 106.
THORNES, R. D. AND MARTIN, W. T.—(1961) *Ir. J. med. Sci.*, **431**, 487.
THORNES, R. D. AND O'MEARA, R. A. Q.—(1961) *Ir. J. med. Sci.*, **428**, 361.